# Editorial: “From brain to body: the impact of nervous system declines on muscle performance in aging”

**DOI:** 10.3389/fnagi.2015.00066

**Published:** 2015-04-30

**Authors:** Brian C. Clark, Timothy D. Law, S. Lee Hong

**Affiliations:** ^1^Ohio Musculoskeletal and Neurological Institute, Ohio UniversityAthens, OH, USA; ^2^Department of Biomedical Sciences, Ohio UniversityAthens, OH, USA; ^3^Department of Geriatric Medicine, Ohio UniversityAthens, OH, USA; ^4^Department of Family Medicine, Ohio UniversityAthens, OH, USA

**Keywords:** muscle, sarcopenia, dynapenia, aging, frailty, weakness, motor control

Around 30% of women and 15% of men in the United States over 60 years self-report that they are unable to lift or carry 10 pounds, and ~50% of women and 40% of men report difficulty in stooping, crouching, or kneeling (Louie and Ward, 2010). Further, more than 40% of seniors have limitations in performing one or more daily tasks (e.g., walking two to three blocks, transferring from sitting to standing) that are essential for maintaining physical independence (Louie and Ward, 2010). While many factors contribute to reductions in physical function, one contributor is skeletal muscle impairments (e.g., muscle weakness) (Manini et al., 2007). While the nervous system is widely recognized for its role in coordination, its role in determining the performance characteristics of aged skeletal muscle has largely been understudied.

Historically, it was believed that the reductions in muscle performance were primarily resultant of age-associated adaptations in skeletal muscle (e.g., muscle atrophy). However, the vast range of motions and forces that humans can achieve arises from the activity of more than 600 skeletal muscles, which are under the control of the nervous system. As such, the nervous system is in all likelihood a critical contributor to all aspects of aged-related changes in muscle performance, and as a consequence, motor behavior (Rosso et al., 2013). Indeed a growing body of research indicates that a good predictor of impending cognitive decline in older adults is a slowed and stooped gait, which has been assigned the term “motoric cognitive risk syndrome” (Verghese et al., 2013, 2014a,b). In this *Frontiers in Aging Neuroscience* research topic, we solicited articles on a broad range of issues surrounding: (1) the age-related changes in nervous system anatomical, physiological, and biochemical changes in the central and/or peripheral nervous systems; (2) the functional role of these nervous system changes in contributing to altered skeletal muscle performance and/or mobility; and (3) the physical and pharmacologic interventions that act via the nervous system to enhance muscle performance and/or mobility. We invited individuals, both *via* invitation and an open call for manuscripts, engaged in aging, neuroscience, and/or applied physiology research focused within the scope of this research topic, to contribute an original research article, review article, clinical case study, hypothesis and theory article, method article, opinion article, or technology report.

In this issue we present 12 articles within this scope. Specifically, in this issue we present 2 review articles, 1 theory article, and 9 original research articles. Below we highlight some of the most notable findings from this research topic issue:

- A general theme at the level of brain activation that arises is the dedifferentiation and compensatory activation. Coppi et al. (2014) and McGregor et al. (2013) both found increased interhemispheric interactions and decreased interhemispheric inhibition in older adults. Similarly, Heetkamp et al. (2014) found older adults to exhibit more diffuse, bilateral brain activation patterns during unilateral motor tasks. In the theory and hypothesis paper by Sleimen-Malkoun et al. (2014), the concept of differentiation in aging is co-constructed with the loss of complexity framework, presenting an argument that these patterns of decline are inherent at the level of brain, muscle, and behavior. In a similar vein, Berchicci et al. (2014) showed that exercise reduces the amount of brain activation needed to perform cognitive tasks in older adults, and Yao et al. (2014) reports that older adults require greater activation in higher-order cortical fields for controlling eccentric muscle contractions.
- Another general theme that arises in the Vanden Noven et al. (2014) and Hasson and Sternad (2014) original articles is that increasing task complexity in older people results in increased motor variability. Of particular interest was the finding from Vanden Noven et al. (2014) indicating that motor performance was dramatically impaired when a high demand cognitive task was performed concomitant with the motor function task. Findings of this nature have implications for injury risk reduction approaches as well as ergonomic applications.
- Two original articles by scientists from the University of Florida examined the neural contributors to mobility in older people. The first of these, from Cruz-Almeida et al. (2014) indicates that sensory tactile perception at the first metatarsal head was associated with both usual and maximal walking speed, while the second article by Clark et al. (2014) observed that the changes in activity of the prefrontal cortex during performance of complex walking tasks were linked to the quality of gait in older adults. Additionally, one of the review articles from Iosa et al. (2014) discusses mobility issues associated with aging, specifically the decline in upright gait stability. They point out that the loss of skeletal muscle with aging, even in healthy older adults, contributes to the inability to maintain an upright posture during walking along with decreased sensory and cognitive declines. A confluence of these declines, they argue, contributes to increased instability and risk of falls.
- The last of the articles, a review by Gonzalez-Freire et al. (2014), points out that aging and its associated loss of muscle mass and strength, are related to neuromuscular junction dysfunction. As a result, they postulate that interventions such as exercise and calorie restriction can positively affect the neuromuscular junction.

Overall, the articles within this Research Topic point to an intimate relationship between brain and nervous system function and its impact of muscle activation, and consequently, motor behavior. Physical fitness and exercise seem to be a central component in maintaining both brain and nervous system health, as well as motor function, against the effects of aging.

## Conflict of interest statement

The authors declare that the research was conducted in the absence of any commercial or financial relationships that could be construed as a potential conflict of interest.
